# Cognitive and Behavioral Abnormalities in FTD Mutation Carriers: Insights into Shared Mechanisms with ADD/ADHD Disorder

**DOI:** 10.1002/alz70857_105621

**Published:** 2025-12-25

**Authors:** Yara Alkhodair, Hyunwoo Lee, Ian R MacKenzie, Emily Dwosh, Howard J. Rosen, Brad F. Boeve, Adam L. Boxer, Ging‐Yuek Robin Hsiung, the ALLFTD Consortium

**Affiliations:** ^1^ Division of Neurology, Department of Medicine, University of British Columbia, Vancouver, BC, Canada; ^2^ Djavad Mowafaghian Centre of Brain Health, Vancouver, BC, Canada; ^3^ Department of Pathology, University of British Columbia, Vancouver, BC, Canada; ^4^ Vancouver Coastal Health, Vancouver, BC, Canada; ^5^ UBC Hospital Clinic for Alzheimer Disease and Related Disorders, Vancouver, BC, Canada; ^6^ Department of Medical Genetics, Faculty of Medicine, University of British Columbia, Vancouver, BC, Canada; ^7^ Department of Neurology, Memory and Aging Center, University of California San Francisco, San Francisco, CA, USA; ^8^ Department of Neurology, Mayo Clinic, Rochester, MN, USA; ^9^ Memory and Aging Center, Department of Neurology, Weill Institute for Neurosciences, University of California, San Francisco, San Francisco, CA, USA

## Abstract

**Background:**

Frontotemporal dementia (FTD) is a neurodegenerative disorder marked by early impairments in executive function and altered prefrontal neural circuitry. These traits are also observed in Attention Deficit Disorder (ADD) and Attention Deficit Hyperactivity Disorder (ADHD), suggesting the possibility of shared mechanisms and a role for developmental neurocognitive factors in the onset of FTD. High‐penetrance mutations in GRN, C9orf72, and MAPT increase FTD risk and are suspected to influence neurodevelopment.

This study hypothesizes that pre‐symptomatic carriers of these mutations exhibit cognitive and behavioral traits that overlap with those observed in ADD/ADHD.

**Method:**

This cross‐sectional study analyzed participants from the ARTFL‐LEFFTDS Longitudinal FTLD (ALLFTD) Study. Inclusion criteria restricted the sample to mutation carriers with pathogenic variants in GRN, C9orf72, or MAPT while excluding individuals with prior psychiatric diagnoses (e.g., bipolar disorder, major depressive disorder) and those taking antipsychotic or anxiolytic medications to avoid potential confounding effects on test validity and interpretation. The final sample included *N* = 338 participants, categorized as pre‐symptomatic mutation carriers (*N* = 227) or asymptomatic noncarriers (*N* = 111).

To assess potential overlaps with ADD/ADHD traits, a targeted subset of measures reflecting executive function, working memory, attention, social behavior, and hyperactivity were extracted from validated cognitive and neuropsychiatric assessments. Mutation carriers were further classified as either converters (*N* = 20) who progressed to symptomatic FTD (CDR ≥ 1) or non‐converters (*N* = 95) who remained asymptomatic. Only pre‐conversion scores were analyzed for converters. Group comparisons were conducted using general linear models adjusted for age and sex.

**Result:**

Pre‐symptomatic mutation carriers showed significantly lower verbal fluency (*p* = 0.004) and a trend toward reduced working memory (*p* = 0.05) compared to noncarriers. Converters exhibited poorer overall cognitive performance (*p* <0.0001), verbal fluency (*p* = 0.001), and memory and attention (*p* = 0.04) compared to non‐converters. Converters demonstrated higher Neuropsychiatric Inventory scores (*p* = 0.0001), particularly in hyperactivity (*p* = 0.003), reflecting greater behavioral disturbances.

**Conclusion:**

Baseline impairments in cognitive function, memory, and neuropsychiatric profiles distinguish converters from non‐converters, highlighting overlaps between ADD/ADHD traits and prodromal FTD. These findings suggest shared neurobiological mechanisms and underscore the need for mutation‐specific studies to further explore the neurodevelopmental factors driving FTD progression.

